# The In Ovo Delivery of CpG Oligonucleotides Protects against Infectious Bronchitis with the Recruitment of Immune Cells into the Respiratory Tract of Chickens

**DOI:** 10.3390/v10110635

**Published:** 2018-11-15

**Authors:** Upasama De Silva Senapathi, Mohamed Sarjoon Abdul-Cader, Aruna Amarasinghe, Guido van Marle, Markus Czub, Susantha Gomis, Mohamed Faizal Abdul-Careem

**Affiliations:** 1 Faculty of Veterinary Medicine, University of Calgary, Health Research Innovation Center, 3330 Hospital Drive NW, Calgary, AB T2N 4N1, Canada; yaseshwari.desilvase@ucalgary.ca (U.D.S.S.); mohamedsarjoon.moham@ucalgary.ca (M.S.A.-C.); arunavetlk@gmail.com (A.A.); vanmarle@ucalgary.ca (G.v.M.); m.czub@ucalgary.ca (M.C.); 2 Department of Veterinary Pathology, Western College of Veterinary Medicine, University of Saskatchewan, Saskatoon, SK S7N 5B5, Canada; smg127@mail.usask.ca

**Keywords:** In ovo, CpG oligonucleotide, infectious bronchitis virus, avian macrophage, CD4+ cell, CD8α+ cell

## Abstract

The in ovo delivery of cytosine-guanosine (CpG) oligodeoxynucleotides (ODNs) protects chickens against many bacterial and viral infections, by activating the toll-like receptor (TLR)21 signaling pathway. Although the delivery of CpG ODNs in ovo at embryo day (ED) 18 has been shown to reduce infectious bronchitis virus (IBV) loads in embryonic chicken lungs pre-hatch, whether in ovo delivered CpG ODNs are capable of protecting chickens against a post-hatch challenge is unknown. Thus, our objectives were to determine the protective effect of the in ovo delivery of CpG ODNs at ED 18 against IBV infection encountered post-hatch and, then, to investigate the mechanisms of protection. We found significantly higher survival rates and reduced IBV infection in the chickens following the pre-treatment of the ED 18 eggs with CpG ODNs. At 3 days post infection (dpi), we found an increased recruitment of macrophages, cluster of differentiation (CD)8α+ and CD4+ T lymphocytes, and an up-regulation of interferon (IFN)-γ mRNA in the respiratory tract of the chickens. Overall, it may be inferred that CpG ODNs, when delivered in ovo, provide protection against IBV infection induced morbidity and mortality with an enhanced immune response.

## 1. Introduction

Infectious bronchitis (IB) is mainly an acute and severe disease of the respiratory system of chickens [1]. The causative agent, infectious bronchitis virus (IBV), belongs to the family *Coronaviridae* [2]. There is increasing evidence of IBV infection being reported in birds other than chickens [3,4]. Although IBV induced changes are observed primarily in the mucosal surfaces of the respiratory tract, the virus is also known to cause pathology in the female reproductive tract and kidneys, with a varying degree of severity dependent upon the type of strain that infects and replicates in the aforementioned tissues [5,6,7].

Ever since the first record of IB in the early 1930s [8], periodic IB outbreaks associated with the isolation of heterogeneous strains of IBV have been reported globally [1,9]. Major losses to the broiler meat industry are due to carcass condemnation at processing, a poor feed conversion ratio resulting in poor weight gain, and mortality. IBV is considered a highly infectious agent with near 100% morbidity, and with mortality reaching 10–25% [10,11]. In breeder and layer flocks, the major losses are due to reduced egg production during and after infection with IBV. The egg drop during IBV infection has been estimated to be between 3–50% [11]. Furthermore, the downgrading of eggs because of a poor internal egg quality and egg shell quality also account for considerable production losses [12]. The standard preventive measures, such as strict quarantine and biosecurity measures [13], do not seem to sufficiently control the disease. Thus far, the most efficient method for controlling IBV is by vaccination [14]. The immunization of chickens against IBV is mainly by live attenuated and killed vaccines [15]. However, the emergence of variant IBV strains/serotypes arising from vaccinated flocks, among other factors, has led to vaccine failure and IB outbreaks. Thus, the development of novel approaches as an alternative or adjunct in order to control the current measures against IBV is becoming increasingly important.

Toll-Like receptor (TLR)s are a family of germ line encoded pattern recognition receptors (PRRs) expressed on the surface or within the endosomal compartments of cells [16]. These receptors are crucial for recognizing whole or segments of microbial pathogens, and they initiate key host immune defenses against inciting agents. Among the TLRs, TLR9 (in mammals)/TLR21 (in birds) are the only receptors capable of distinguishing bacterial, parasitic, and viral DNA containing cytosine-guanosine (CpG) motifs [17]. Several studies have demonstrated the immunostimulatory and therapeutic success of CpG oligodeoxynucleotides (ODNs) application in various host–pathogen interaction models [18,19,20]. The protection provided by CpG ODNs against lethal challenges of extracellular bacteria, such as *Escherichia coli* [18] and *Salmonella* Typhimurium [19], and viruses, such as low pathogenic avian influenza virus [20] and infectious laryngotracheitis virus (ILTV) [21,22], in chickens have been well documented. CpG ODNs are known to induce an array of cytokines; chemokines; and effecter molecules, such as interferon (IFN) α, β and γ, interleukin (IL)-1β, IL-6, IL-12, IL-8, tumor necrosis factor (TNF)-α, and nitric oxide (NO) [23,24,25]. These effecter molecules are believed to play a pivotal role in protecting the host against intra and extra cellular pathogens. Whilst activating a variety of immune cells, it plays an integral role in bridging the innate immune system with the adaptive immune system directing immune responses toward T helper (Th)1 response [26].

A study that pre-treated chicken embryos with class B CpG ODNs at embryo day (ED) 18 in ovo, and then challenged them with IBV Ark99 strain the day after (ED 19), showed an increased up-regulation of IFN-γ, IL-1β, IL-6, IL-8, and oligoadenylate synthetase (OAS) A in the embryonic spleen [23]. Additionally, the authors saw a significant reduction in the IBV *nuclear (N)* gene mRNA expression in various embryonic tissues pre-treated with CpG ODNs, compared with the control, highlighting the value of CpG ODN treatment in IBV control. However, they did not demonstrate whether the in ovo CpG ODN delivery is effective against the IBV challenge encountered post-hatch. In this study, we determined whether the CpG ODNs delivered in ovo could provide protection against a post-hatch IBV challenge. Furthermore, we looked into several cytokines and immune cells that may be activated with such protection. We found that in ovo delivery was protective against IBV challenge post hatch, suggesting a potential lasting protective effect of CpG ODNs towards IBV infection, which could be exploited for developing control measures.

## 2. Materials and Methods

### 2.1. Animals

The specific pathogen free (SPF) eggs from white leghorn layer hens were obtained from the Canadian Food Inspection Agency (CFIA), Ottawa, and were incubated according to the manufacturer’s instructions in digital egg incubators (Kingsuromax 20 and Rcom MARU Deluxe max, Autoelex Co., Ltd., GimHae, GyeongNam, Korea), located at the Health Research Innovation Centre (HRIC) 53, University of Calgary. All of the animal care protocols as well as the use of live chickens, embryos, and SPF eggs in our experiments, have been reviewed and approved by the Health Science Animal Care Committee (HSACC, AC14-0013, 20 November 2014). At ED 11, the incubated eggs were candled in order to select viable eggs for further incubation, and the hatched birds were transported and housed in high containment poultry isolators at the Prion/virology animal facility, HRIC, University of Calgary, with access to ad libitum food, water, and necessary veterinary care.

### 2.2. Virus, Virus Propagation, and Titration

The IBV Massachusetts (M)41 strain was purchased from the American Type Culture Collection (ATCC, Manassas, VA, USA) and was used in all of the experiments. Nine day old SPF viable eggs were used to propagate M41 strain of IBV, and the allantoic fluid was harvested at 3 dpi by careful aspiration. The end point dilution assay was employed to assess the viral titers using ED 9 SPF eggs, and was expressed as a 50% embryo infectious dose (EID_50_) [27].

### 2.3. TLR Ligand

The synthetic CpG ODNs, class B CpG motifs recognized by chicken TLR21 (5′-TCG TCG TTG TCG TTT TGT CGT T-3′), and the negative control ODN 2007 (5′-TGC TGC TTG TGC TTT TGT GCT T-3′) were purchased from Cedarlane (Burlington, ON, Canada), and were used in our experiments.

### 2.4. Experimental Design

#### 2.4.1. Assessment of Protection Provided by the in Ovo Delivery of CpG ODNs against Post-Hatch IBV Infection

Fifty µg of class B CpG ODN 2007 was diluted in 200 µL of phosphate buffered saline (PBS), and was inoculated per SPF egg on ED 18, via the in ovo route (*n* = 7). The control ODNs were diluted in PBS to the same concentration (50 µg in 200 µL per egg), and were delivered via the same route (*n* = 6). The in ovo TLR ligand delivery was carried out as described previously [20]. On day 1 post-hatch, the birds in both groups were infected with IBV M41 strain intra-trachealy, at a dose rate of 2.75 × 10^4^ EID_50_ per bird, and were monitored for 11 days post-infection (dpi) for disease progression and outcome. The humane end point of the birds was determined based on the clinical score of each bird (ruffled feathers and huddling together = 1, droopy wings = 1, depression = 1, mild increase in respiratory rate = 1, increased respiratory rate with constant beak opening =2, severe increased respiratory rate marked by gasping = 3, and body weight loss = 1). The clinical score of 5 was considered the humane endpoint.

#### 2.4.2. Determination of Mechanisms of in Ovo Delivered CpG ODN-Induced Protection against IB

In order to evaluate the mechanisms of protection of the in ovo delivered CpG ODNs, the ED 18 SPF eggs were injected with class B CpG ODN 2007 (50 µg diluted in 200 µL of PBS, *n =* 21), control ODNs (50 µg diluted in 200 µL of PBS, *n =* 29), and PBS alone (200 µL, *n =* 11), as described earlier. The eggs were then incubated for 3 days until hatching. On the day of hatching, a subset of CpG ODN-treated chickens (*n =* 12) were infected with IBV M41 strain intra-trachealy at a dose rate of 2.75 × 10^4^ EID_50_ per bird, while maintaining the rest of the birds in that treatment group as uninfected controls (*n =* 9). Similarly, a subset of the control ODN-treated chickens was infected with IBV M41 strain with the same dose (*n =* 18), with the remaining birds being the control ODN treated birds (*n =* 11), and all the PBS treated birds (*n =* 3) were kept as controls. The birds were weighed, wing tagged and after infection, were placed in separate isolators until the subsets of the animals were euthanized at 3 (*n =* 5–9 per group) and 7 (*n =* 3–9 per group) dpi. The clinical signs were observed and recorded daily as described, and the oro-pharyngeal and cloacal swab samples obtained using Puritan^®^UniTranz-RT^®^ Media Transport Systems (VWR, Edmonton, AB, Canada) at 3 and 7 dpi, and the IBV genome load were quantified following the RNA extraction. Simultaneously, the lung tissue was collected at 3 and 7 dpi in RNA Save^®^ (Biological Industries, FroggaBio, Toronto ON, Canada), in order to determine the viral genome loads in lungs. To evaluate the IBV N antigen in the tracheal mucosal epithelium, the tracheal tissues from the 3 dpi birds were collected and preserved in an optimum cutting temperature (OCT) compound (Tissue-Tek^®^, Sakura Finetek USA inc, Torrance, CA, USA), and were snap frozen in dry ice until use in immunofluorescent assay. To observe the histopathology, the tracheal tissues of the 3 dpi birds were fixed in 10% neutral buffered formalin (VWR International, West Chester, PA, USA) and sent to the Histopathology Diagnostic Services Unit at the University of Calgary, Faculty of Veterinary Medicine, for hematoxylin and eosin (H and E) staining. Additionally, the trachea and lung tissues were collected in an OCT compound (Tissue-Tek^®^, Sakura Finetek USA inc, Torrance, CA, USA), snap frozen, and subjected to immunofluorescent assay so as to quantify the key innate and adaptive immune cells. Another portion of the tissues were collected iusing RNA Save^®^ (Biological Industries, FroggaBio, Toronto ON, Canada) for the cytokine mRNA expression analysis. The animal numbers represent the total number of animals in two independent experiments.

#### 2.4.3. RNA Extraction, Complementary (c)DNA Conversion, and Real Time Reverse Transcriptase Polymerase Chain Reaction (RT-PCR) Assay

The total RNA from the lungs collected at 3 and 7 dpi was extracted using a Trizol reagent (Invitrogen, Canada Inc., Burlington, ON, Canada), according to the manufacturer’s guidelines. For the RNA extraction of oro-pharyngeal and cloacal swabs, the E.Z.N.A.^®^ viral RNA kit (Omega Bio-tek Inc., Norcross, GA, USA) protocol was adopted as per manufacturer’s guidelines. The concentration of extracted RNA was measured using Nanodrop1000 spectrophotometer (ThermoScientific, Wilmington, DE, USA), with the absorbance at a 260/280 nm wavelength. Two µg of total RNA from the tissue samples and 200 ng of total RNA from the swab samples were used to synthesize the cDNA with the use of the High Capacity cDNA Reverse Transcription Kit (Invitrogen Life Technologies, Carlsbad, CA, USA), as per manufacturer’s guidelines.

A RT-PCR assay was carried out using Fast SYBR^®^ Green Master Mix (Invitrogen, Burlington, ON, Canada) in order to quantify the IBV *N* gene and cytokine mRNA expressions. RT-PCR assays were conducted in a 96 well un-skirted, low profile PCR plate (VWR, Edmonton, AB, Canada), where the final reaction volume of the qPCR was maintained at 20 µL. Each qPCR run consisted of samples of interest, a positive control/s (gene specific plasmid), negative reverse transcriptase (NRT) control (cDNA construct without the multiscribe reverse transcriptase enzyme), and negative template (NTC) control. All of the cDNA samples originating from the tissues, along with the plasmid dilution series used to generate the standard curves, were run in triplicate. The target genes were quantified in relation to the *β actin* housekeeping gene. The target gene and the housekeeping gene for each sample was run on the same plate. Five picomolar (pM) of different gene specific primers (Forward and Reverse primers) were used in each reaction (Supplementary Table S1). The change in the mRNA expression of the cytokines was assessed using the Pfaffl method [28]. The optimum parameters used in the Thermal Cycler (CFX96-C1000) (Bio-Rad Laboratories, Mississauga, ON, Canada) were 95 °C for 20 seconds (s) of pre-incubation, 95 °C for 3 s, and 60 °C for 30 s for 40 amplification cycles. A melting curve analysis was performed between 95 °C and 65 °C, with a 0.5 °C raise in temperature every 5 s. The acquisition of fluorescent signals was performed at 60 °C for 30 s.

#### 2.4.4. Immunofluorescent Assay

For the cluster of differentiation (CD)8α+ cell and macrophage (KUL01+) of the lung and trachea, 5 μm thick sections were cut from the OCT preserved tissues and were fixed using cold acetone for 5 minutes (min). The tissues were then blocked by adding 5% goat serum diluted in a Trizma buffered saline (TBS) buffer (Trizma base: 2.42 g; NaCl: 8 g in 1 L of distilled water; pH 7.6) at room temperature for 30 min. After tipping off the excess blocking buffer, as the primary antibodies, the mouse monoclonal antibody specific for chicken macrophages/monocytes, KUL0+ (Southern Biotech, Birmingham, Alabama, USA), CD8α (CT-8, Southern Biotech, Birmingham, Alabama, USA), was used in a 1:200 dilution in a 5% goat serum for 30 min. The secondary antibody, goat anti-mouse IgG (H+L) conjugated with Dylight^®^ 550 (red fluorescence) (Bethyl Laboratories Inc., Montgomery, TX, USA) was then used in a 1:500 in 5% goat serum for 1 hour (h), followed by adding Vectashield^®^ mounting medium with 4′, 6-Diamidine-2′-phenylindole dihydrochloride (DAPI, Vector Laboratories Inc., Burlingame, CA, USA) (Blue fluorescence), placing cover slips and edges sealed with lacquer as the final step.

For the CD4+ T cell staining, before blocking the tissues with 5% goat serum, sections were blocked with avidin followed by biotin (Vector Laboratories, Inc., Burlingame, CA, USA), each with 15 min incubation periods, in between washing with TBS-T for 3 min twice and with PBS for 3 min once. After blocking with 5% goat serum for 30 min, a primary antibody, CD4 (CT-4, Southern Biotech, Birmingham, Alabama, USA) was added in a 1:200 dilution in 5% goat serum for 30 min. Next, biotinylated goat anti-mouse IgG (H+L) (Southern Biotech, Birmingham, Alabama, USA) was used as a secondary antibody in a 1:250 dilution in a 5% blocking buffer, and was incubated for 30 min. Then, DyLight^®^ 488 (green fluorescence) streptavidin in a 15:1000 dilution was added for 30 min, followed by a final step of mounting the slides with a Vectashield^®^ mounting medium with DAPI (Vector Laboratories Inc., Burlingame, CA, USA). All of the incubations were performed in a humidifying chamber at room temperature. Each incubation with an antibody was followed by washing the slides in a TBS-T buffer for 3 min twice and in PBS for 3 min once.

### 2.5. Data Analyses

For the quantification of the tissue KUL01+, CD4+ cells, and CD8α+ cells, five areas with maximum positive fluorescent signals of KUL01+, CD4+ cells, and CD8α+ cells per tissue section were captured at X 20 magnification, along with the corresponding nuclear stained (DAPI) areas. The images were then subjected to fluorescent intensity quantification using Image J software (National Institute of Health, Bethesda, MD, USA). The fluorescent intensities for the Dylight^®^ 550 (KUL01+, CD8α+ cells) and DyLight^®^ 488 (CD4+ cells) positive signals were expressed relative to the total area (as estimated by nuclear staining with DAPI), and were given as a percentage.

### 2.6. Statistical Analyses

A log-rank test was used to identify the differences in survival percentage. The Kruskal–Wallis test followed by the Mann–Whitney U test were used to identify the group differences in the clinical score data for each time point. The differences among the two groups were identified using othe student’s *t* test. One-way analysis of variance (ANOVA) followed by the Students–Newman–Keuls test were used to identify the group differences in all of the other experiments. The Grubbs’ outlier test was performed in order to identify the outliers before the data was analyzed. The data in the graphs are shown in the original scale of the measurements. However, because of the non-normality and inability to satisfy the model assumptions of data belonging to the cell counts and cytokine mRNA expression, a natural log transformation was applied to these data sets prior to analysis. Model statistics were performed using GraphPad Prism Software 5, La Jolla, CA, USA. A normality test, generation of histograms, box plots, and Q–Q plots were performed in R statistical software, R studio version 1.0.153, Boston, MA, USA. * = significant at *p* ≤ 0.05, ** = significant at *p* ≤ 0.01 *** = significant at *p* ≤ 0.001.

## 3. Results

### 3.1. In Ovo Delivery of CpG ODNs is Protective Against IBV Infection Encountered Post-Hatch

**Figure 1:**
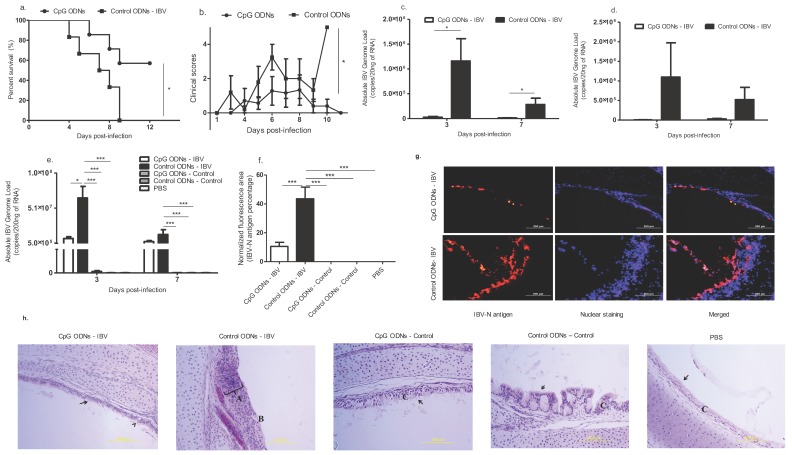
In ovo delivery of cytosine-guanosine (CpG) oligodeoxynucleotides (ODNs) is protective against infectious bronchitis virus (IBV) infection encountered post-hatch. Specific pathogen free (SPF) embryo day (ED) 18 eggs were delivered in ovo with class B CpG ODNs (*n* = 7) or control ODNs (*n* = 6), and on day 1 post-hatch, the chickens were infected with IBV M41 strain at a dose rate of 2.75 × 10^4^ embryo infectious dose (EID_50_) per bird, and were monitored until 11 days post infection (dpi). (**a**) Survival percentage and (**b**) clinical scores. (**c**–**h**): SPF ED 18 eggs were delivered in ovo with class B CpG ODNs (*n* = 21), control ODNs (*n* = 29), and phosphate buffered saline (PBS) (*n* = 11). The eggs were incubated until hatching, and on day 1 post-hatch, a subset of in ovo CpG ODN-treated birds was challenged with IBV M41 strain at a dose rate of 2.75 × 10^4^ EID_50_ per bird (*n* = 12), and the rest were kept as in ovo CpG pre-treated uninfected controls (*n* = 9). Similarly, a subset of birds in the in ovo control ODN-treated birds was infected with IBV (*n* = 18), and the remaining birds were kept as in ovo control ODN-treated uninfected controls (*n* = 11). The in ovo PBS treated birds were kept as uninfected controls (*n* = 11). A subset of birds from each group was sacrificed at 3 dpi (*n =* 5-9 per group), and the remaining birds were sacrificed at 7 dpi (*n =* 3–9) in order to obtain lung tissue. (**c**) IBV genome loads in oro-pharyngeal swabs at 3 and 7 dpi, (**d**) IBV genome loads in cloacal swabs at 3 and 7 dpi, and (**e**) IBV genome loads in 3 and 7 dpi lung. (**f**–**g**) The quantitative data and representative figures from the immunofluorescent assay of the trachea for IBV N antigen is presented. Scale Bar = 200 μm (**h**) Representative images of histological observations of trachea are given. Control ODNs – IBV: Severe epithelial metaplasia with severe cellular infiltration, germinal center formation is seen (A), superficial epithelial layer has become squamous with complete loss of cilia (B) and mucus glands not detected. CpG ODNs-IBV: pseudostratified simple columnar epithelium and intact ciliated epithelia (arrow) is evident where some have become rounded, and a few mucus secreting glands have been distorted and elongated (arrow head). CpG ODNs-control, Control ODNs-control, and PBS-control: No lesions, normal pseudostratified ciliated columnar epithelium (C) with mucus secreting glands. Log-rank test was used to identify the differences in the survival rate, and the Kruskal–Wallis test followed by the Mann–Whitney U test were used to identify the differences in the clinical scores at selected time points. The student’s t test was performed to identify group differences in the oropharyngeal and cloacal genome loads, and one-way analysis of variance (ANOVA) followed by the Students–Newman–Keuls post hoc test was used to identify the differences in the lung IBV genome loads and IBV N antigen amount in the trachea. The differences were considered significant at * = significant at *p* ≤0.05, ** = significant at *p* ≤0.01 *** = significant at *p* ≤0.001. c–h: the animal numbers and results represent the pooled data of the two independent experiments.

We observed a significant increase in the survival rate of the CpG ODN-treated chickens (*p* < 0.05) when compared with the control ODN-treated chickens, as seen in Figure 1a. Also, the clinical signs in the CpG ODN-treated IBV-infected group were significantly milder compared with the control ODN-treated and IBV-infected group at 10 dpi (*p* < 0.05, Figure 1b).

In order to assess the IBV genome loads in the lungs, and to determine the degree of virus shedding through the feco-oral route, the IBV *N* gene was quantified at 3 and 7 dpi in the birds that were pre-treated with in ovo CpG ODNs, control ODNs, and PBS. We observed a significant reduction in the viral genome loads in the oro-pharyngeal swabs collected 3 and 7 dpi (*p* < 0.05; Figure 1c), but did not observe a difference in the IBV genome loads in the cloacal swabs (3 and 7 dpi) between the treatment groups (*p* > 0.05; Figure 1d). However, significantly lower levels of lung viral genome load in the in ovo CpG ODN pre-treated IBV infected group compared to in ovo control ODN pre-treated IBV-infected group were observed at 3 dpi (*p* < 0.0001; Figure 1e). At 7 dpi, although the control ODN pretreated IBV infected lung had a significantly higher IBV genome load when compared with the uninfected controls (*p* < 0.0001; Figure 1e), the difference of the IBV genome load in the lungs between two IBV infected groups was not significant (*p* > 0.05; Figure 1e).

We observed a significant reduction of the IBV-N antigen in the tracheal mucosal epithelium of the in ovo CpG ODN pre-treated-IBV infected birds compared with the in ovo control ODN pre-treated-IBV infected birds (*p* < 0.0001; Figure 1f–g). This was also seen in the histology of the trachea for the degree of tracheal damage following IBV infection. The mucosal epithelium of the in ovo control ODN treated-IBV infected group showed severe metaplasia with severe mononuclear cell infiltration, where the superficial epithelial layer had been replaced by squamous cells. A complete erosion/loss of the entire mucosae was evident in several areas of the trachea. Also, mucus secreting glands were absent from the remaining mucosae. In contrast, in the in ovo CpG ODN pre-treated-IBV infected birds, the epithelium was a pseudostratified simple columnar epithelium mostly with intact cilia on the surface. Mucus glands were present with some distortion and elongation (Figure 1h).

### 3.2. In Ovo Delivery of CpG ODNs is Capable of Recruiting Key Cells of the Innate and Adaptive Arms of the Immune System Responsible for Enhanced Immune Responses in the Respiratory Tract

**Figure 2:**
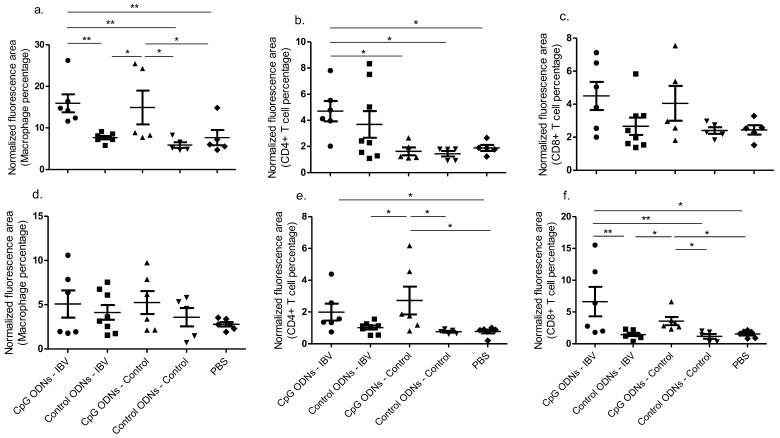
In ovo delivery of CpG ODNs is capable of recruiting key cells of the innate and adaptive arms of the immune system responsible for enhanced immune responses in the respiratory tract. The quantitative data following immunofluorescent assays done for the trachea (**a**) macrophages, (**b**) cluster of differentiation (CD)4+ T cells, and (c) CD8α+ T cells are given. The quantitative data following the immunofluorescent assays done for lung (**d**) macrophages, (**e**) CD4+ T cells, and (**f**) CD8α+ T cells are given. One-way ANOVA followed by the Students–Newman–Keuls post hoc test were used to identify the group differences. The differences were considered significant at * = significant at *p* ≤ 0.05, ** = significant at *p* ≤ 0.01 *** = significant at *p* ≤ 0.001. The results represent the pooled data of two independent experiments.

In general, the in ovo CpG ODN pre-treated IBV infected group recorded higher macrophage and CD4+ and CD8α+ T numbers in the trachea compared with the uninfected and control ODN pre-treated groups, although not all of the specific comparisons reached statistical significance (Figure 2a–c). Similarly, the in ovo CpG ODN pre-treated IBV infected group recorded higher macrophage and CD4+ and CD8α+ T numbers in lungs compared to uninfected and control ODN pre-treated groups, although not all of the specific comparisons reached statistical significance (Figure 2d–e). In the trachea and lungs, the macrophage and CD8α+ T recruitment patterns, respectively, indicated that the in ovo delivered CpG ODNs are capable of increasing the recruitment of these cells in both the IBV infected and uninfected chickens (Figure 2a–e; *p* < 0.01).

### 3.3. In Ovo Delivery of CpG ODNs is Capable of Inducing Pro-Inflammatory Mediator mRNA Expressions in Lungs

**Figure 3:**
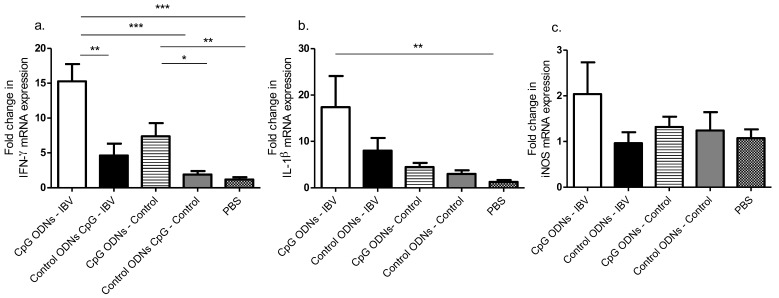
In ovo delivery of CpG ODNs is capable of inducing pro-inflammatory mediator mRNA expression levels in the lungs. (**a**) Fold change in the interferon (IFN)-γ mRNA expression, (**b**) fold change in the IL-1β mRNA expression and **c**) iNOS mRNA expression of 3 dpi lung. One-way ANOVA followed by Students-Newman-Keuls post hoc test was used to identify differences in mRNA expression levels. The differences were considered significant at * = significant at *p* ≤ 0.05, ** = significant at *p* ≤ 0.01 *** = significant at p ≤ 0.001. The results represent pooled data of two independent experiments.

Considering that we observed a significant reduction in the IBV induced morbidity and mortality of in ovo CpG ODN pre-treated birds correlating with varying degrees of increased macrophages, CD4+, and CD8α+ T cells in the tracheal and lung tissues, we needed to further elucidate the mechanisms by which these immune cells were efficiently recruited. Several cytokine mRNA expression levels in the lungs were analyzed at 3 dpi, and our data showed a significant increase in the up-regulation of only the IFN-γ mRNA expression in the in ovo CpG ODN pre-treated lungs compared with the in ovo control ODN pre-treated lungs, in both the IBV infected (*p* < 0.01) and uninfected (*p* < 0.05) groups (Figure 3a–c).

## 4. Discussion

In ovo delivery of poultry vaccines has been performed routinely for decades by the poultry industry [29]. In ovo delivery targets the deposition of CpG ODNs in the amniotic cavity. Subsequently, the ingestion of CpG ODNs containing amniotic fluid by the developing embryo distributes CpG ODNs in the respiratory and gastrointestinal tracts, leading to immune cell recruitment in these two body systems [22]. We have shown in this study that CpG ODNs when delivered in ovo are capable of protecting young chickens against a post-hatch IBV infection induced IB. In ovo CpG ODN-treated birds displayed reduced IBV viral loads in the lungs and a decreased IBV replication and pathology in the trachea, which is associated with high survival rates and low morbidity. We found that the macrophages in the trachea and CD4+ and CD8α+ T cells in the lungs play important roles in this process, as increases in these cells were observed in the in ovo CpG ODNs treated group. Lastly, we saw an up-regulation of IFN-γ mRNA in the in ovo CpG ODN pre-treated lungs, suggesting the critical role of this cytokine in the in ovo CpG ODN-induced clearance of IBV infection.

The host survival after day 1 post-hatch IBV infection was seen as significant in the presence of CpG ODN administration in ovo compared with the controls, and a similar protective effect of in ovo delivered CpG ODNs has been recorded against the post-hatch ILTV infection [21,22], and *E. coli* and *Salmonella* Thypimurium septicemia [18,19]. In the current study, the protection-mediated by the in ovo administered CpG ODNs was associated with significantly lower IBV replication in the trachea and IBV genome loads in the lungs at 3 and 7 dpi. Consequently, the IBV genome loads in the oro-pharyngeal swabs were significantly reduced at 3 and 7 dpi. However, we did not observe a significant reduction in the IBV genome loads in the cloacal swabs at 3 and 7 dpi, because of the high variability of the IBV genome loads within the control ODN pre-treated group. It is difficult to explain why we observed a discrepancy in the IBV genome loads between the oro-pharyngeal and cloacal swabs, as the in ovo delivered CpG ODNs have been shown to recruite immune cells into the gastrointestinal mucosa [22].

Our data confirm that, when delivered in ovo, the CpG ODNs are able to recruit macrophages into the trachea at 3 dpi (four days of age), when compared to the in ovo delivered control ODNs in both the IBV infected and uninfected groups. Previously, we saw that the in ovo delivered CpG ODNs increased the macrophages in the trachea at one day of age [21,22]. This macrophage recruitment to the trachea is associated with a lower IBV replication in the tissue, and it is possible that the CpG ODN-mediated increase of the macrophages seen in the trachea in this study, played a central role in limiting the viral replication by three possible mechanisms. First, these cells may have efficiently and rapidly phagocytized the virus-infected cells and aided in virus elimination. Second, they may have alerted the adaptive immune system to the invasion via active antigen presentation to the T cells. Third, it may have contributed to the T and B cell activation and proliferation through the release of cytokines [30,31].

Our observation of the increased recruitment of CD4+ and CD8α+ T cells in the lungs following in ovo CpG ODNs delivery indicated that CpG ODNs could act as a mitogen, as has been shown previously [32]. This CpG ODN-mediated increased CD4+ and CD8α+ T cell recruitment also could be due to the increased survival of these T cells in the lungs [32]. Interestingly, we saw an expansion of the CD8α+ T cell population, but not the CD4+ T cell population in the in ovo CpG ODN pre-treated-IBV infected lungs. Although a portion of this increase of CD8α+ cell recruitment could be potentially attributable to the IBV specific CD8+ T cells, we did not determine whether these CD8α+ T cells are in deed IBV specific. We are at a loss as to why we did not see a similar CD4+ and CD8α+ T cell response in the trachea, but it is possible that the in ovo delivered CpG ODN-mediated CD8α+ T cell recruitment is tissue specific [22]. It is also important to note that our data is limited to 3 dpi, and we do not know whether the in ovo delivered CpG ODN-mediated CD4+ and CD8α+ T cell recruitments in the trachea are occurring in other time points.

Of the examined immune mediators, IFN-γ, a dominant product of the T helper (Th)1 type cells, was upregulated in the CpG ODN pre-treated IBV infected and uninfected groups, when compared with the control ODN pre-treated IBV infected and uninfected groups. The source of the lung IFN-γ mRNA could be the CD4+ T cell lymphocytes and CD8α+ cytotoxic lymphocytes [33,34], and we observed an increased recruitment of the CD4+ and CD8α+ T cells in the lungs in our experiment.

Two other immune mediators that were induced by the CpG ODNs and originated from the innate immune cells, such as the macrophages in the lungs, are the chemoattractant, IL-1β, and the NO production inducer, iNOS [21]. In the current study, we did not observe that the CpG ODNs or IBV induced the mRNA expression of IL-1β or iNOS. This discrepancy in the CpG ODN-mediated lack of IL-1β and iNOS expression can be explained by the difference in the time points observed. Thapa et al. [21] observed an increase IL-1β mRNA expression in the lungs pre-hatch, and we observed a lack of IL-1β mRNA expression post-hatch following in ovo CpG ODNs delivery.

The significance of the observations described in our study are two-fold. First, we found that the in ovo administration of CpG ODNs is capable of limiting IBV replication in the lungs and trachea, leading to an increased survival and reduced morbidity in the early post-hatch birds. Second, in our study, the early recruitment and maintenance of key immune cells, such as CD8α+ and CD4+ T cells and macrophages, and the up-regulated IFN-γ mRNA, exhibited not only an initiation of the early innate response, but also an effective and early adaptive host response mediated by the CpG ODNs, which would facilitate protection against the IBV infections encountered in birds in their immediate post-hatch life.

Further experiments elucidating the mechanisms of the CpG ODN-mediated adoptive response, such as cell- and antibody-mediated immune responses in chickens and the duration of protection provided by this ligand against IBV, would be greatly beneficial in order to better understand the protective effects of CpG ODNs, and may aid in the development of more effective IBV control measures.

To conclude, we show that the CpG ODN-mediated protective response against post-hatch encountered IBV infection is associated with the up-regulation of IFN-γ mRNA expression (in the lungs) and the enhanced recruitment of macrophages (in trachea) and CD4+ and CD8α+ T cells (in the lungs). Our findings, although preliminary, may provide a basis for developing novel control strategies in the long term against IBV infection in chickens.

## Supplementary Materials

The following are available online at http://www.mdpi.com/1999-4915/10/11/635/s1, Table S1: PCR primers used in real time PCR techniques.
