# Supplementary material for: The In Ovo Delivery of CpG Oligonucleotides Protects against Infectious Bronchitis with the Recruitment of Immune Cells into the Respiratory Tract of Chickens
Source: Viruses. 2018 Nov 15;10(11):635. doi: 10.3390/v10110635 (PMC6266937; doi:10.3390/v10110635)
Supplement: Supplementary file 1 [file viruses-10-00635-s001.pdf]

Supplementary Table 1. PCR primers used in real time PCR techniques.

Primer	Sequence (5′-3′)	Fragment (bps)	Reference
IBV-N	F-GACGGAGGACCTGATGGTAA<br>R-CCCTTCTTCTGCTGATCCTG	206	[2]
β-actin	F-CAACACAGTGCTGTCTGGTGGTA<br>R-ATCGTACTCCTGCTTGCTGATCC	205	[2]
IL-1β	F-GTGAGGCTCAACATTGCGCTGTA<br>R- TGTCCAGGCGGTAGAAGATGAAG	214	[2]
iNOS	F-GGCAGCAGCGTCTCTATGACTTG<br>R-GACTTTAGGCTGCCCAGGTTG	185	[2]
IFN-γ	F-ACACTGACAAGTCAAAGCCGCACA<br>R-AGTCGTTCATCGGGACCTTGGC	129	[2]
